# Oligodendrocytes and Microglia: Key Players in Myelin Development, Damage and Repair

**DOI:** 10.3390/biom11071058

**Published:** 2021-07-20

**Authors:** Ilias Kalafatakis, Domna Karagogeos

**Affiliations:** 1 Laboratory of Neuroscience, Department of Basic Science, University of Crete Medical School, 70013 Heraklion, Greece; ilias_kalafatakis@imbb.forth.gr; 2 IMBB FORTH, Nikolaou Plastira 100, Vassilika Vouton, 70013 Heraklion, Greece

**Keywords:** oligodendrocytes, microglia, myelin, inflammation, demyelination, remyelination, multiple sclerosis

## Abstract

Oligodendrocytes, the myelin-making cells of the CNS, regulate the complex process of myelination under physiological and pathological conditions, significantly aided by other glial cell types such as microglia, the brain-resident, macrophage-like innate immune cells. In this review, we summarize how oligodendrocytes orchestrate myelination, and especially myelin repair after damage, and present novel aspects of oligodendroglial functions. We emphasize the contribution of microglia in the generation and regeneration of myelin by discussing their beneficial and detrimental roles, especially in remyelination, underlining the cellular and molecular components involved. Finally, we present recent findings towards human stem cell-derived preclinical models for the study of microglia in human pathologies and on the role of microbiome on glial cell functions.

## 1. The Importance of Myelin

The myelin sheath is the membranous structure that surrounds most of the central (CNS) and peripheral (PNS) nervous system axons of vertebrates. It is an evolutionary adaptation that allows fast signal propagation along bigger distances, a feature of the increasing body size of animals. Myelin is produced by myelinating glial cell, the oligodendrocyte in the CNS and Schwann cell in the PNS, spirally arranged around the axon in a compact sheath. Signal conduction is optimal due to the increase in resistance and decrease in the membrane capacitance of axons, which increase the velocity of action potential propagation up to tenfold [1,2].

In addition to this important insulating function, myelin assumes several critical functions in the nervous system. It protects axons mechanically by isolating the electrical signal from the microenvironment. Importantly, oligodendrocytes have been shown to provide metabolic support to neurons and to regulate ion and water homeostasis [3,4,5,6]. It has been appreciated recently that oligodendrocytes influence neuronal circuits by being able to respond to activity-dependent changes by regulating myelin production [7,8,9,10]. Indeed, our understanding of the events leading to myelin production has been enriched by recently identified regulatory factors that control different aspects of glial development and by large scale analysis showing a great diversity and complexity of progenitors and mature cells involved in myelination, a process that is seen as active throughout the life of the organism.

The regulation of myelination, either during development or during repair or adaptive conditions, is mediated by complex interactions between neurons and glia. In particular, all types of glial cells participate in not yet completely elucidated ways, in myelin development and its modulation. In this review we discuss essential aspects of the cross-talk between oligodendrocytes and another glial cell type, the microglia, in maintaining myelin homeostasis, and also examine how this cross-talk is influenced under conditions of inflammation that often lead to myelin pathologies.

## 2. Oligodendrogial Development at a Glance

Myelinating oligodendrocytes originate from oligodendrocyte precursor cells (OPCs) which are highly proliferative cells derived in discrete waves during development. In the forebrain ventricular zone, for example, neuroepithelial progenitors generate OPCs from E12.5 until birth. OPCs are characterized by the expression of platelet-derived growth factor receptor A (PDGFR-a), the neuron-glial antigen 2 (NG2) proteoglycan and gangliosides recognized by the A2B5 antibody. Elegant studies on oligodendroglial development have elucidated the mechanisms of specification and differentiation of OPCs to the myelinating phenotype and have shown that Shh-dependent (early phase) and independent (late phase) signaling, as well as expression of the transcription factors Olig1, Olig2, Mash, Nkx2.2 and Sox10, are essential in these processes. OPCs migrate while still proliferating to populate the CNS, differentiate to a premyelinating identity, and at this point are able to wrap around axons; however, they are still unable to form mature myelin, a step that occurs upon expression of myelic basic protein (MBP) and myelin oligodendrocyte glycoprotein (MOG). Several key regulators of this journey have been identified, including morphogen signaling (Shh, Wnt) as well as several transcription factors and miRNAs [11,12,13].

As myelin keeps being generated in the healthy adult CNS, at least two sources of OPCs have been revealed, namely the progenitors of the subventricular zone (SVZ) and the NG2+/PDGFRα+ cells distributed throughout the CNS. These cells maintain their proliferative ability so that oligodendrocytes are generated continuously. Recent data points to the formation of new oligodendrocytes as an important process in myelin plasticity coupled to the learning of motor skills [14,15].

## 3. Myelin Damage

Demyelination is defined as myelin loss around axons, either due to congenital conditions or a CNS insult (such as inflammation or injury). The prototype of demyelinating pathological entity is multiple sclerosis (MS), a chronic, inflammatory disease causing focal destruction of the myelin sheath and a glial scar. MS is the most common acquired demyelinating CNS disease with yet unknown etiology. In brain magnetic resonance imaging (MRI) the pathological hallmark of the disease manifests itself as white matter plaques that are hyperintense lesions widely scattered throughout the CNS, although the optic nerves, brainstem, spinal cord and periventricular white matter are the sites usually affected. The pathogenesis of MS is characterized by disruption of the blood-brain barrier with subsequent active immune cell recruitment and a cascade of pathologies that range from infiltration of lymphocytes and activation of microglia to the destruction of the myelin sheath and the degeneration of axons [16,17,18].

In MS, a cascade of neuroinflammatory processes finally leads to the injury of myelinating oligodendrocytes causing demyelination; although the initial insult leading to oligodendrocyte death and myelin loss remains elusive, these events could arise from infiltrating immune cells but also within the CNS itself [19].

It is generally accepted that a prophagocytic activity precedes full-blown myelin phagocytosis. These early events do not show evidence of oligodendrocyte apoptosis, pointing to alternative mechanisms of cell death. Microglia are observed in these early-stage lesions. Myelin phagocytosis is considered to arise by the action of myelin-specific T cells that have been activated by antigen-presenting cells (microglia and dendritic cells) and thus start the full inflammatory sequence of events. In the previously resting inflammatory cascade, parenchymal microglia also participate by releasing cytokines such as interleukin 12 (IL-12) and IL-13, which enhance Th1 cell differentiation and myelin damage through the release of NO and glutamate. Using an inducible model of adult oligodendrocyte depletion, it was shown that oligodendrocyte loss is followed by CD4+ T cells leading to a secondary demyelination [20]. Although some of the pathophysiological mechanisms and players responsible for CNS demyelination have been described [21], the full picture still needs to be elucidated. Thus, it is unclear whether oligodendrocyte injury is the root or the outcome of inflammation.

Several models of demyelination in rodents exist. Not a single one is able to recapitulate all aspects of human disease, but the most common models resemble distinct phases and stages of the condition (inflammatory, autoimmune and demyelination-remyelination processes). In these models, demyelination is brought about through immune system stimulation, local or systemic toxin introduction, genetically-encoded toxin expression or viral transduction. The experimental autoimmune encephalomyelitis (EAE), the toxic cuprizone demyelination model and the lysolecithin (LPC) model are among the most commonly used [22,23].

## 4. Myelin Repair

Myelin repair occurs in the human CNS as well as in some rodent models of MS. Remyelinated axons have a characteristically thin myelin sheath and short internodes. However, extensive heterogeneity exists due to disease duration, stage and phenotype. It is unclear at the moment whether indeed, as some studies show, remyelination is more efficient in early demyelinating lesions and then follows a diminishing course [24,25]. It has been observed that the demyelinating disease progression superimposes and takes over myelin repair, indicating a close interplay between the two processes [26] suggesting myelin repair is not restricted to early phases of demyelinating pathology.

Why then, is repair after CNS insult not sustained, as evidenced by the majority of MS lesions being of the chronic demyelinating type [27]? The reasons for remyelination failure are not well understood [28]. It was shown that axons do not become properly remyelinated due to the formation of a glial scar [29,30]. Periventricular lesions seem to be more affected than deep white matter or cortical lesions. A number of factors have been described as possible underlying reasons for limited myelin repair over the course of disease; among them, OPC numbers may be decreased [31,32]. In MS, as well as in mouse models, OPC recruitment and differentiation to mature myelinating oligodendrocytes is negatively affected [33,34]. OPCs gradually lose their remyelinating ability and need to be activated so that they can become responsive to factors enhancing their mobilization to the injured area and proceeding to differentiate to the myelinating phenotype [35,36,37,38,39]. OPC differentiation and recruitment is negatively impacted by an unfavorable microenvironment due to increased expression of Notch and Wnt signaling, as well as modulation of expression of chemorepellent Semaphorin 3A and chemoattractant Semaphorin 3F [34,40,41]. Another factor that negatively affects remyelination is aging. Aging leads to changes of the microenvironment that negatively affect OPC recruitment and differentiation during demyelinating conditions [42].

Animal models resembling stages of MS, as well as studies on postmortem human tissue, show that adult progenitors with OPC characteristics, as well as neural stem cells expressing glial fibrillary acidic protein (GFAP) and Nestin at the SVZ, are able to remyelinate denuded axons [43,44,45,46]. In general, myelin repair is the outcome of complex interactions between all glial types, not just oligodendrocytes, as is detailed in the following sections. However, the oligodendrocytes are essential players and, as described below, their functions extend well beyond myelin formation.

## 5. Oligodendrocytes: More than Just Forming Myelin

Recently, the intriguing idea that OPCs may be harnessed by the immune system under demyelination conditions to perpetuate the autoimmune response has been put forward [47,48]. OPCs and oligodendrocytes in both human and murine settings are able to act as antigen-presenting cells and activate CD8+ T cells. A subset of oligodendroglial cells express genes involved in antigen processing and presentation and have an inflammatory signature as assessed by single cell RNAseq, are therefore named iOPC/iOLs. These studies reveal an intermediary oligodendrocyte phenotype in humans, while in the mouse both OPC and OL upregulate antigen-presenting molecules [47,49,50]. Therefore, these cells have the potential to be in direct communication with CD8+ T cells. Although many aspects of this novel activity of oligodendroglial cells need to be investigated, experiments using T cells recognizing foreign peptides, and transgenic lines with cell-type specific expression of the foreign peptides, suggest that antigen presentation by iOPC/iOL is effective after T cells have already been primed by dendritic cells. What is interesting, in addition, is the possibility the iOPLC/iOL may be able to modulate aspects of OL survival and differentiation.

One more active area of research regarding the oligodendrocyte microenvironment has been the investigation of exosomes (extracellular vesicles, EVs) in the light of their function as mediators of cross-talk between various cellular players, including oligodendrocytes and microglia [51,52,53,54,55,56,57]. Originally recognized as a mechanism of discarding unwanted cellular material, EVs are also important signaling intermediaries. EVs released by oligodendrocytes may represent a significant vehicle of communication during development and upon injury. Neuronal signals trigger OL-EVs which, in turn, are taken up by neurons or microglia in their microenvironment [51,53,54,55]. In particular, microglia pick up OL-EVs via micropinocytosis, and subsequently process the contents though the endo-lysosomal compartment and thus maintain the homeostatic turnover of myelin components. OL-EVs may also mediate neuroprotection by providing support to neurons upon demand. Similar studies in pathological contexts may be helpful in elucidating the role of OL-EVs in disease. An autocrine model of OL-EV mode of action has been suggested to operate in inhibiting myelin formation in vitro [58]. In this context, it was recently shown that EVs released from activated microglia block remyelination, a process mediated directly by astrocytes, while EVs released from anti-inflammatory microglia promoted remyelination in a lysolecithin-induced demyelinating lesion ([59] and relevant section below).

## 6. Microglia: Origins and Homeostatic Functions

Microglia comprise 10% of total glial cells and represent the myeloid resident population of the CNS [60]. Microglia originate from yolk-sac macrophages (YSM) entering the brain after the first generation of neurons [61], while expanding and self-renewing during adulthood [62]. Apart from their immune roles, recent studies have shown that both fetal and adult microglia contribute to diverse processes including synaptic transmission, synaptic pruning and formation, cell death and survival, as well as brain wiring [62,63].

In the 100 years since they were first established as a distinct CNS population, research on this versatile cell type has revolutionized the field of neuroinflammation [64]. Microglia have been traditionally described as the macrophages of the CNS, able to scavenge dying cells, pathogens and molecules, as well as myelin debris in pathological conditions, but more recently they have been recognized as much more than typical scavengers. They are characterized by a continuum of activation states. Microglia are the brain-resident, macrophage-like, innate immune cells that are able to respond with the necessary flexibility in a context-specific nature. Under homeostatic conditions, microglia exhibit a ramified morphology, and their role is to “scan” the CNS for potential insults. In general, microglia are able to switch between pro and anti-inflammatory states in order to maintain tissue homeostasis in response to the environment, and are able to interact with neurons, astrocytes and oligodendrocytes contingent on their particular function [65]. A recent study focused on the analysis of the RNA expression patterns of more than 76,000 individual microglia in mice during development, in old age, and after brain injury. The results showed at least nine transcriptionally distinct microglial states, which expressed unique sets of genes and were localized in the brain using specific markers. The highest heterogeneity was found at young ages, although several chemokine-enriched inflammatory microglia persisted even in the aged brain. Multiple reactive microglial subtypes were also found following demyelination in mice. These results can be used for the better understanding of microglia function and for the identification and manipulation of specific microglia subpopulations in health and disease [66].

## 7. Role of Microglia in Myelin Development

During development, microglia play essential roles in the establishment of neuronal numbers as well as shaping neuronal circuits by clearing dead cells and by pruning or remodeling synapses, and, in general, modulating emerging neuronal wiring [67,68,69,70,71,72,73,74]. In addition to this important array of functions, microglia also support OPCs and OLs. The developing white matter contains microglial clusters which are thought to be involved in myelin formation [75,76]. One possible way microglia can be involved in developmental myelination is by secreting trophic factors for which OPCs, generated in excess, compete [77]. During development, activated microglia of the SVZ secrete TNFα, IL-1β, IL-6 and IFN-γ, and promote oligodendrocyte development. When the levels of these cytokines are reduced, oligodendrogenesis is impaired [78]. Early work on microglia-oligodendrocyte cocultures indicated that microglia activate oligodendrocytes to synthesize the myelin-specific galactolipid sulfatide in addition to myelin proteins MBP and PLP [79].

A series of in vitro studies have supported multiple mechanisms of microglia beneficial roles on OPC survival and maturation [80,81,82]. Microglia-conditioned medium rescues OPCs from cell death due to growth factor withdrawal and promotes their differentiation [83]. Similar media enhance OPC chemotaxis and proliferation. IL-2 and IL-10 activation of microglia enhances survival and differentiation of OPCs [84]. IL-4 activation of microglia drives neural precursors towards the oligodendroglial lineage [85]. In vivo studies of a knockout of *CSFIR*, a gene encoding a tyrosine kinase receptor required in macrophages, showed a significant depletion of microglia, perturbed brain development and a severe decrease in mature oligodendrocytes in particular areas of the CNS [86,87]. As transcriptome analysis of CNS region-specific microglia suggests, microglia populations in distinct brain areas may be more or less supportive of oligodendroglial development [88]. Single cell profiling studies have revealed a great degree of regional heterogeneity of microglial populations with the cerebellar one having a unique profile [89,90,91]. An example of a unique population of microglia that mainly appears in primary myelinating areas of the developing brain is the CD11c+ microglial subset that expresses genes for neuronal and glial survival, migration and differentiation. CD11c+ microglia have an amoeboid morphology and localize close to white matter tracts (corpus callosum and cerebellum). They are present early during embryonic development, while their numbers peak between P3 and P7. Upregulation of 39 genes is associated with developmental CD11c+ microglia, revealing a common signature for this specific cell type. These cells constitute the major source of insulin-like growth factor 1 (IGF-1), while their selective depletion leads to impairment of primary myelination [76].

As mentioned above, microglia phagocytose neurons and synapses during development. A recent study revealed that they also phagocytose excess myelin sheaths, a process regulated by neuronal activity and called myelin pruning [92]. Another study showed that a specific population of microglia migrate from the ventricular zone into the corpus callosum during development, phagocytosing viable OPCs before the onset of myelination. Mice lacking the fractalkine receptor exhibit a reduction in microglial engulfment of viable OPCs, increased numbers of oligodendrocytes and reduced myelin thickness, indicating that microglia phagocytose OPCs as a homeostatic mechanism leading to proper myelination. Impairment of microglial pruning of OPCs could lead to hypomyelinating developmental disorders or adult demyelinating diseases [93].

In the adult, contrary to their active role in development, microglia have been considered rather inactive. However, in vivo imaging studies have revealed that microglial cells actively survey their surroundings [94,95] and interact with their neighbors [96]. Factors regulating microglia morphology in adult brain homeostasis include purinoreceptors, ion channels and neurotransmitters [97,98]. Microglia clearing of cell debris does not require them to be in an activated (amoeboid) state.

## 8. Role of Microglia in Demyelinating Disorders

In the past few years it has become evident that microglia are active determinants of neurodegenerative diseases, among them Alzheimer’s, Huntington’s, Parkinson’s disease and MS [64]. This review focuses on the involvement of microglia in MS and, in particular, the interactions of microglia and oligodendrocytes either in the context of MS or in animal models resembling aspects of MS. In these pathological conditions, microglia are shown to release both pro and anti-inflammatory soluble factors, and present self-antigens to immune cells.

During CNS injury, microglia are activated, changing to an amoeboid morphology. When microglia are activated they can either promote inflammation and cytotoxicity by secreting factors such as IL-1, IL-12, IL-23, tumor necrosis factor alpha (TNFa) and inducible nitric oxidase synthase (iNOS) or reduce inflammation and promote neuroprotection by secreting factors such as IL-4, IL-10, IL-13, transforming growth factor beta (TGFβ) and arginase 1 (Arg-1) [87,99,100].

Microglia are involved in both the de and remyelination phase of MS [101]. During demyelination, microglia undergo phagocytosis of myelin debris and secrete proinflammatory molecules [102,103,104,105]. Postmortem analysis of lesions in MS patients shows a high number of immature oligodendrocytes in acute, active lesions, which are characterized by a robust inflammatory response suggesting that extensive oligodendrocyte regeneration occurs in some plaques early in the course of the disease [106]. It is thus considered that the inflammatory environment during demyelination may stimulate the process of remyelination, starting with the myelin debris clearance by microglia [107,108,109,110]. Following injury, different inflammatory factors are secreted by microglia in the surrounding milieu such as IL-12, IL-13, NO, TNFα and glutamate. The net result is antigen-presenting activity and cytokine/chemokine release on the part of microglia propagating inflammation within the CNS. Intriguingly, this microglial activation and accumulation within the damaged area of the neuronal parenchyma plays both beneficial and detrimental roles during myelin damage and repair.

## 9. Beneficial Roles of Microglia

**Figure 1:**
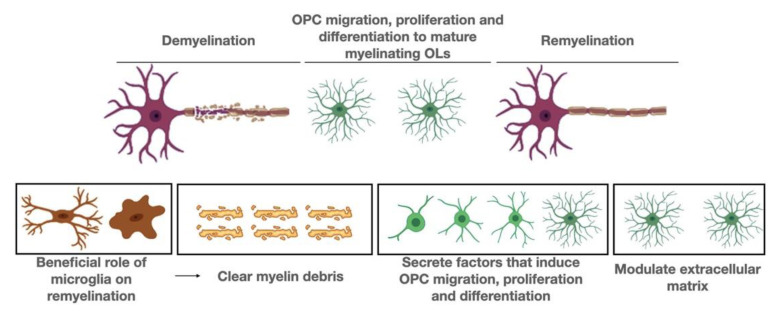
Beneficial role of microglia on remyelination. Microglia promote remyelination in three ways. First, they remove myelin debris, a procedure that is essential for the initiation of remyelination. Second, they secrete factors that promote OPC migration, proliferation and differentiation into mature myelinating oligodendrocytes. Third, they modify the extracellular matrix.

It has been demonstrated beyond doubt that neuroinflammation promotes neurogenesis and facilitates axonal regeneration and remyelination. In this section we focus on molecular pathways that may be operating in microglial cells, with particular emphasis on remyelination (Figure 1).

Conditioned medium from nonactivated microglia increased OPC survival and differentiation by upregulating the PDGFRα-signaling pathway accompanied by NF- kB activation [80]. The iron status of microglia also affects OL survival, as iron is a cofactor for enzymatic activity in CNS cells and OLs, which are demanding in energy needs and particularly sensitive to iron dysregulation. Conditioned medium from iron-loaded microglia increased survival of OL cultures via the release of H-ferritin, although iron-loaded LPS-activated microglia had the reverse effect [111]. Insulin growth factor 2 (IGF-2), present in conditioned media from nonactivated and activated microglia also promoted OPC survival and differentiation [80].

In vitro studies show that microglia-conditioned medium accelerated OL differentiation in a more efficient manner than conditioned-medium from astrocyte cultures. Analysis of the media revealed a distinct composition in the two types of media. Astrocyte-conditioned medium contained higher quantities of platelet-derived growth factor alpha (PDGFα), fibroblast growth factor 2 (FGF2), FGF2-binding protein, ciliary neurotrophic factor (CNTF), growth hormone (GH), TIMP metallopeptidase inhibitor 1 (TIMP-1) and thrombospondin (THBS1). In contrast, in microglia-conditioned medium, levels of IGF-1, E-selectin (CD62E), fractalkine (CX3CL1), neuropilin-2 (NRP-2), IL-2, IL-5 and vascular endothelial growth factor (VEGF) were significantly increased. This differential composition of cytokines and growth factors in the conditioned media of astrocytes and microglia reflects the distinct intracellular signaling pathways that are activated in OPCs upon exposure to the two different media [83].

Another chemokine that is expressed by microglia and influences the OL lineage is CXCL-1. It reduces OPC migration during development, while it protects OLs from apoptosis during viral-induced demyelination [112,113]. Additionally, CXCL-1 overexpression reduces the severity of EAE [114].

During remyelination, microglia can play both beneficial and detrimental roles. Microglia in early remyelinating lesions in MS patients are characterized by a predominant expression of the chemokine receptor CCR5, indicating that microglia may play a role in the initiation of remyelination [115]. A change in microglia polarization from an activation to a repair profile can initiate remyelination [84]. In this seminal work, in vitro, ex vivo and in vivo data indicate that M2 microglia (the anti-inflammatory type) drive OL differentiation and remyelination. As remyelination starts, a switch from M1 (proinflammatory) microglia phenotype to an anti-inflammatory phenotype is evident. M2 depletion inhibits OL differentiation in vivo, and increased M2 numbers are seen during efficient remyelination. Oligodendrocyte differentiation is enhanced with M2 microglia-conditioned medium in vitro and impaired with M1-conditioned medium. Furthermore, the authors showed that activin-A in the M2-derived media is able to drive OL differentiation in ex vivo cerebellar organotypic slices [84].

A well-established aspect of microglia during remyelination concerns their fundamental role in the clearance of myelin debris. Myelin debris has to be removed from the injury site in order for the remyelination process to be effective [116]. In the cuprizone-induced model of demyelination, myelin debris clearance by microglia depends on the presence of the microglial triggering receptor expressed on myeloid cells 2 (TREM2). TREM2 is expressed on the cell surface and binds polyanions, thus activating downstream signaling cascades through the adapter DAP12. Trem2(-/-) microglia fail to amplify transcripts indicative of activation, phagocytosis, and lipid catabolism in response to myelin damage, and thus Trem2(-/-) mice cannot efficiently phagocytose myelin debris, a necessary step to promote remyelination [87,112]. In the same toxic demyelination cuprizone model, it was shown that astrocytes recruit microglia to the lesion site to clear damaged myelin, a process which is regulated by the chemokine CXCL-10. Upon depletion of astrocytes and, consequently, microglia, removal of myelin debris is significantly delayed, resulting in the inhibition of OPC proliferation and resulting in decreased remyelination [117]. With age, microglia become less efficient at clearing myelin debris, while they also alter their gene expression profiles. Astrocytes also change during aging and it is possible that these changes have a negative impact on remyelination, although additional studies should be performed [42]. In a recent study it was shown that not only myelin removal by microglia is essential for remyelination, but also myelin proper degradation. Defective metabolism of myelin leads to cholesterol accumulation intracellularly in microglia cells, resulting in activation of the inflammasome, increase of the inflammatory reaction and blockage of remyelination [118].

During remyelination, microglia express galectin-3 (Gal-3), an essential factor for OPC differentiation [82,119]. Another molecule that positively affects OLs is osteopontin (OPN). Previous studies showed that OPN, expressed by microglia, both increased OPC proliferation and myelin synthesis in vitro [120]. CXCL-12 (otherwise known as stromal cell-derived factor 1), a factor well studied and shown to be important in OPC migration, is expressed by microglia in MS lesions [121,122]. Microglia surrounding MS lesions are shown to express Semaphorin-3F (SEMA3F), which is able to attract OPCs to damaged areas [123].

A surprising finding regarding the role of TNF-α, known to be secreted by activated microglia as a pro-inflammatory cytokine (see also next section), has been described [124]. In the cuprizone mouse model, lacking TNF-α and its associated receptors led to a delay in remyelination. This failure of myelin repair was correlated with reduced OPC progenitors and an associated reduction of mature OLs [124]. Moreover, it was revealed that when TNFR2, but not TNFR1, was missing, regeneration was impaired. Thus, TNF-α has a reparative role other than its more well-known detrimental function. Other signaling molecules that have been shown to be important in vivo in the clearance of myelin debris and remyelination include IL-1β [29] and macrophage colony-stimulating factor (M-CSF), which stimulates survival and differentiation of myeloid cells [87,125]. Finally, Arg-1 is a key factor in the regulation of microglia activation, as this enzyme is expressed by microglia upon internalization of myelin and is correlated with suppression of neuroinflammation [84,126]. In EAE, when Arg-1 expression is enhanced, the progression of the disease is reversed [127]. However, inhibition of Arg-1 may be correlated with impaired immune responses and thus the role of Arg-1 remains unclear [128].

Apparently, both M1 and M2 microglia have a function in remyelination, because in early stage, M1 (pro-inflammatory) phenotypes predominate while at later post demyelination stages the M2 phenotypes are more abundant. When the corresponding types (M1 or M2) are depleted, OPC differentiation is impaired [84,129]. Mononuclear phagocytes, including microglia, play an important role in both tissue damage and repair in neuroinflammatory conditions such as MS. A recent study introduced an in vivo imaging approach that allowed the temporal and spatial investigation of the evolution of phagocyte polarization in a murine model of MS. In this study it was shown that individual phagocytes shift their phenotype in response to CNS-derived signals. These findings suggest that the initial proinflammatory polarization of phagocytes, including microglia, needs to be prevented rather than reversed. Moreover, the timing of interventions aiming to block phagocyte infiltration or activation is crucial. While these interventions are possibly beneficial during lesion formation, they could have opposing effects during lesion resolution [130].

Human MS brain studies corroborate the idea that microglia contribute in a beneficial manner to the process of remyelination. The density of microglia correlates with the density of OPCs and, in addition, a higher density of microglia at the lesion borders of plaques correlates with higher remyelination propensity [131,132].

Many of the studies on microglial activation have been performed by incubating the cells with lipopolysaccharide (LPS). In these studies, microglia promote OPC and OL survival through IGF-1 and the PI3/Akt pathway as well as the mitochondrial apoptotic pathway due to suppression of caspase activity [133]. However, in vivo treatment with IGF-1 in a rodent EAE model failed to provide conclusive evidence of its efficacy [134,135]. In vitro LPS treatment with microglia showed an increase in the levels of expression of neurotrophins, supporting the idea that proinflammatory microglia are able to express neuroprotective molecules [136]. In particular the role of BDNF has been studied in detail and shown that by producing it, microglia impair antigenic presentation and promote not only neuronal but also OL survival/differentiation in rodent de and remyelination models such as the cuprizone model [137,138,139,140,141,142].

## 10. Detrimental Roles of Microglia

**Figure 2:**
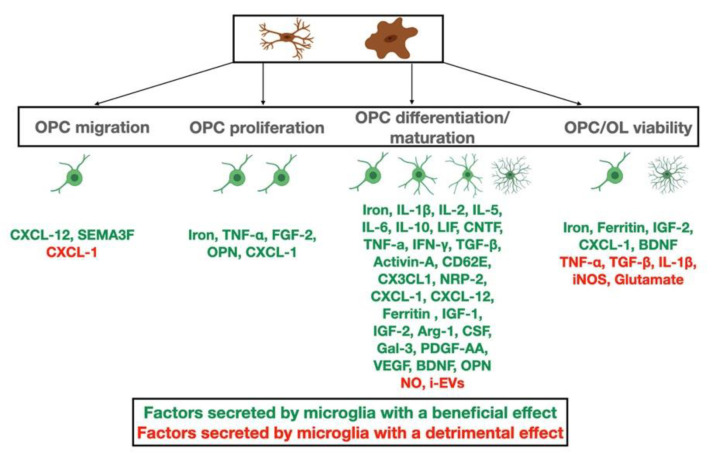
Inflammatory factors secreted by microglia during demyelination and remyelination that affect OPC migration, proliferation and differentiation to mature myelinating OLs, as well as OPC/OL survival. Factors with beneficial effects are listed with green color, while factors with detrimental effects are listed with red color.

As discussed above, the view that proinflammatory microglia have damaging effects on neurogenesis or gliogenesis is outdated. Evidence has been mounting that inflammation not only aims at clearing debris but also at promoting tissue regeneration. However, there are several detrimental effects of microglia that we discuss below (Figure 2).

Microglia are able to exert antigen presenting functions on myelin-specific T cells infiltrating the CNS, thus initiating inflammation. As a result, resting microglia start secreting Th1-promoting cytokines IL-12 and IL-13. Th1 cells, in turn, release a number of agents toxic to myelin, including NO and glutamate [143,144]. If these microglia are selectively eliminated, EAE symptoms become less severe and myelin loss is reduced, while blockade of CD40, which is involved in the activation process, also reduces EAE severity [145,146]. Activated microglia secrete TNFα, a strong proinflammatory cytokine; when this release is blocked EAE symptoms are ameliorated [147]. TAK1, a MAP3K, TGF-β-activated kinase, when inactivated, specifically in microglia, suppress EAE symptoms, via NF-κB, JNK and ERK1/2 pathways. Thus, these pathways seem to be essential for the CNS inflammation and accompanying myelin and axonal damage, mediated by microglia [148].

When microglial cells are stimulated in vitro with LPS, cytotoxic effectors are released and damaging effects on OPCs and OLs are exerted. OPC differentiation is impeded by NO-dependent oxidative damage early on, and by TNFα later on [149]. When astrocytes are present, LPS-activated microglia become toxic to differentiating oligodendrocytes via TNF signaling, but not via NO-dependent oxidative damage [150]. When microglia are polarized by CD137 (a member of the tumor necrosis factor receptor family), OL apoptosis is promoted [151].

The harmful effects of microglia on oligodendrocytes may be mediated through astrocytes due to the release of IL-1α, TNF and C1q [152,153,154,155]. A recent report shows that, in an LPC-model in vivo, EVs released by activated, proinflammatory microglia (i-EVs) block remyelination by blocking OPC differentiation [59]. Through high-resolution EM analysis, the authors observed microglial cells along with oligodendrocytes and astrocytes at the lesion site. The microglia had the appearance of the reactive type with surface protrusions suggesting EV release, which prompted the authors to investigate the exogenous EVs produced by activated microglia with a Th1 cytokine cocktail (IL-1β, TNFα, IFNγ). However, in vitro, proinflammatory-induced microglial EVs were able to directly exert prodifferentiating functions on cultured OPCs; therefore, the authors postulate that the in vivo blockade of remyelination observed may involve the action of other CNS cells, namely astrocytes. The same group showed that microglial EVs were able to transform astrocytes into A1 cells that inhibit OPC differentiation. Analysis of microglial factors able to cause the conversion of astrocytes to the A1 type namely IL-1a, C1q and TNFα, showed that indeed these mediators were present in i-EVs.

## 11. Gray Matter Pathology of Microglia

As discussed above, it is clear that microglia are intimately involved in white matter pathology in multiple ways. On the contrary, their involvement in gray matter pathology has been less studied. A recent, comprehensive review discusses their involvement in myelin pathology and neurodegeneration, highlighting the fact that, in part, microglia in gray matter follow the same principles as in white matter, but also in some ways diverge from them [156].

Gray matter pathology in MS is at the center of the disease, since it is present from the earliest stages and is linked to the disability MS patients suffer from [157]. Microglia are involved in all aspects of gray matter pathology. As evidenced in other degenerative diseases, microglia in gray matter exert both beneficial and damaging functions [158].

In gray matter demyelination, inflammation is present but not as pronounced as in white matter. It is hypothesized, based on histological observations that report inflammatory infiltrates near cortical (in other words gray matter) lesions, that soluble mediators such as interferon-γ and TNF-α, two molecules associated with microglial activation, contribute to demyelination. On the other hand, it is postulated that gray matter demyelination occurs secondarily to white matter demyelination. At present, the exact role of microglia in gray matter is not as clear as in white matter regions.

Chronic gray matter lesions, being less inflammatory than white matter lesions, remyelinate to a greater extent [159]. A number of factors may underlie this important observation. It is possible, for example, that gray matter microglia are more efficient in clearing myelin debris, as they do in development, by eliminating ectopic myelin [92]. At the same time, data from rodent models suggest that in gray matter, OPCs may be assisted by microglia in migrating more efficiently [160]. In addition, studies in iron homeostasis, important for myelin synthesis, point to microglia in gray matter as facilitators in iron intake by oligodendrocytes [161]. Thus, gray matter microglia may be allies in remyelinating efforts in the CNS.

In gray matter pathologies, synaptic function is at stake. In many neurodegenerative diseases, including MS, synaptic compromise appears early. Postmortem studies have revealed microglial engulfment and digestion of synaptic components in cerebellar gray matter, thalamus and hippocampus. Inhibitory synapses seem to be selectively targeted in the motor cortex [162]. Rodent models confirm the human studies [162,163,164].

Other than their damaging effect, however, microglia in gray matter have been shown to protect synaptic health by aiding the survival of synapses to maintain network accuracy [165]. Microglia are essential components of synaptic plasticity, as they induce the generation of postsynaptic structures [166,167].

As neuronal cell bodies, axons and dendrites degenerate, resulting in cell death, and CNS microglia are considered prime culprits. Microglia with a phagocytic phenotype are found apposing neuronal damaged cells, expressing molecules effectuating oxidative stress in demyelinated cortical areas [168,169]. A degeneration-associated microglial signature is present in degenerative pathologies, including MS gray matter disease [170,171]. MS neurodegeneration also involves the action of astrocytes, and cross-talk between microglia and astrocytes is known to contribute [172].

Although a clear role for neuroprotection has not been established for gray matter microglia, some recent evidence in human postmortem tissue, as well as rodent tissue, point to the possibility that they physically protect neuronal cell bodies [173,174].

In summary, microglia in gray matter, although less studied than their white matter counterparts, participate in demyelination, remyelination, synaptic damage and neurodegeneration by supporting both beneficial and detrimental functions on oligodendrocytes and neurons. Thus, as it is gradually appreciated that microglia organize an extremely diverse and multifaceted response, understanding their actions in both white and gray matter is of paramount importance (Table 1).

## 12. Human Stem-Cell-Derived Preclinical Models for the Study of Microglia in Human Pathologies

In rodents, the use of novel transgenic lines is expected to shed light on the role of microglia in the processes discussed above via tracking and manipulation [175,176]. Recent data from single cell analysis also allows in depth characterization of the role of microglia in health and disease. From the limited data so far, regional differences in microglial profiling have emerged in mouse as well as humans [89,170,177]. Although they share a core transcriptome, human microglia seem to be more heterogenous and more immune-vigilant [178]. Thus, the question that human-specific pathologies may not be properly recapitulated or modelled in animals, resurfaces.

It is, therefore, with anticipation that data from the differentiation of human-induced pluripotent stem cells (iPSCs) is projected to elucidate human-related pathology [179,180,181]. These technologies coupled with other state-of-the-art approaches, such as 3D organoids that enable cross-talk between cellular populations, pave a promising pathway. Nevertheless, as in all in vitro models, iPSC-derived microglia are isolated from their microenvironment with unknown consequences. Manipulating human microglia derived from iPSCs, but transplanted in a mouse brain, may provide important clues.

As the ultimate goal is to target microglia for MS therapy, all of the constraints discussed above, namely the regional, temporal and functional heterogeneity that this versatile cellular population exhibits, need to be carefully considered. We still need to reveal aspects of their homeostatic physiology and pathophysiology in order to be able to harness some of their beneficial attributes.

## 13. Microbiome, Microglia and Oligodendrocytes

The emerging field of microbiome research has already provided intriguing information on the relationship of the microbiome and CNS function. In particular, the microbiome-gut-brain axis is shown to play a significant role in neuroinflammation. Functional studies have linked the microbiome to a number of neurodegenerative diseases including MS [182,183,184,185,186]. MS patients possess a distinct microbiome, and, in turn, the microbiome of MS patients induces disease in rodents [183,187,188]. Microglia (as well as other innate immune cells) express toll-like and other pattern recognition receptors that are able to detect microbial and other molecules associated with infections. Upon recognition, microglia release proinflammatory cytokines. Thus, microbe-induced inflammation could play a role in autoimmunity and inflammatory demyelination seen in MS or MS-like pathologies in animals [102]. Gut microbiota could contribute to MS pathogenesis by priming myelin-reactive T cells, as has been observed in EAE models [189]. Prompted by the fact that in MS patients a reduction of bacteria producing short fatty acids, in particular butyrate, in a recent work butyrate was administered to cuprizone-treated mice as well as to LPC-treated organotypic slices resulting in amelioration of demyelination in both cases. Moreover, butyrate seems to affect oligodendrocytes directly without microglial involvement, and it also promoted differentiation of immature oligodendrocytes [190]. Maternal administration of probiotics decreased IL-1β-induced inflammation and was associated with decreased microglial activation, while OPC development was promoted [191]. Recent work on the other hand, does not support a role of microbiota in modulating endogenous CNS remyelination as assessed by OPC differentiation [192]. More mechanistic studies are necessary to establish a direct role of the microbiome in the process of myelination and associated pathologies, although the exciting progress in this field holds great promise.

## 14. Concluding Remarks

In conclusion, homeostasis of CNS myelination is contingent upon the cross-talk between oligodendrocytes, microglia and astrocytes (astrocytes are not discussed in this work but for comprehensive reviews see [172,193]). In the various demyelinating pathologies, this cross-talk is essential in all phases of disease manifestation (inflammation, demyelination, remyelination) regulating the functions of each cellular population. Moreover, these interactions are pleiotropic and dynamic in space and time. Therefore, it is crucial to understand the cellular and molecular nature of the complex dynamics of such interactions, which show both positive and negative functions during damage and repair.

A number of unresolved issues regarding this intricate interplay of microglia and oligodendrocytes in health and disease await careful investigation. Among them, the clarification of the conditions driving microglia towards their beneficial or damaging roles, especially in gray matter of demyelinating disease models, is paramount. Single cell analysis has already been useful, and will continue to be so, in deciphering context-specific genetic programs underlying microglia and oligodendrocyte functions. Research directions that are important in myelin repair, such as neuronal activity, the microbiome as well as the exploitation of human-derived stem cells, are promising. The hope is that knowledge on the modulation of beneficial versus detrimental roles of microglia will be used to devise therapeutic strategies with an emphasis on oligodendrocyte proliferation/maturation.

## Figures and Tables

**Table 1:** Summary table summarizing the functions of microglia during myelin damage and repair and indicating their beneficial or detrimental roles, as well as their source.

Function of Microglia	Beneficial or Detrimental Effect	Samples Used in the Study	White or Gray Matter
Increase OPC survival	Beneficial	In vitro studies	Both
Increase OPC differentiation	Beneficial	In vitro studies	Both
Increase OL differentiation	Beneficial	In vivo studies (cuprizone induced demyelination model)	White matter
Increase OPC proliferation	Beneficial	In vitro studies	Both
Increase OL survival	Beneficial	In vitro studies	Both
Increase OL survival	Beneficial	In vivo studies (cuprizone induced demyelination model)	White matter
Reduce OL apoptosis	Beneficial	In vivo studies (viral induced demyelination model)	White matter
Responsible for myelin clearance	Beneficial	In vivo studies (cuprizone induced demyelination model)	White matter
Responsible for myelin degradation	Beneficial	In vivo studies (LPC induced demyelination model)	White matter
Enhance remyelination	Beneficial	In vivo studies (cuprizone induced demyelination model)	White matter
Initiate inflammation	Detrimental	In vivo studies (EAE demyelination model)	White matter
Enhance disease symptoms	Detrimental	In vivo studies (EAE demyelination model)	White matter
Increase toxicity leading to impaired OL differentiation	Detrimental	In vitro studies	Both
Increase the efficiency of OPC migration	Beneficial	In vivo studies	Gray matter
Facilitate iron intake by oligodendrocytes	Beneficial	Human studies	Gray matter
Engulf and digest synaptic components	Detrimental	Human and in vivo studies	Gray matter
Protect synaptic health	Beneficial	In vivo studies	Gray matter
Essential for synaptic plasticity	Beneficial	In vivo studies	Gray matter
Contribute to neuronal cell body, axonal and dendrite degeneration	Detrimental	Human and in vivo studies	Gray matter
